# The click is not the trick: the efficacy of clickers and other reinforcement methods in training naïve dogs to perform new tasks

**DOI:** 10.7717/peerj.10881

**Published:** 2021-02-22

**Authors:** Rachel J. Gilchrist, Lisa M. Gunter, Samantha F. Anderson, Clive D.L. Wynne

**Affiliations:** Department of Psychology, Arizona State University, Tempe, AZ, USA

**Keywords:** Dogs, Clicker training, Secondary reinforcer, Behavioral conditioning, Reinforcement learning

## Abstract

**Background:**

A handheld metal noisemaker known as a “clicker” is widely used to train new behaviors in dogs; however, evidence for their superior efficacy compared to providing solely primary reinforcement or other secondary reinforcers in the acquisition of novel behavior in dogs is largely anecdotal.

**Methods:**

Three experiments were conducted to determine under what circumstances a clicker secondary reinforcer may result in acquisition of a novel behavior more rapidly or to a higher level compared to other readily available reinforcement methods. In Experiment 1, three groups of 30 dogs each were shaped to emit a novel sit and stay behavior of increasing duration with either the delivery of food alone, a verbal stimulus paired with food, or a clicker with food. The group that received only a primary reinforcer reached a significantly higher criterion of training success than the group trained with a verbal secondary reinforcer. Performance of the group experiencing a clicker as a secondary reinforcer was intermediate between the other two groups, but not significantly different from either. In Experiment 2, three groups of 25 dogs each were shaped to emit a nose targeting behavior and then perform that behavior at increasing distances from the experimenter using the same three methods of positive reinforcement as in Experiment 1. No statistically significant differences between the groups were found. In Experiment 3, three groups of 30 dogs each were shaped to emit a nose-targeting behavior upon an array of wooden blocks with task difficulty increasing throughout testing using the same three methods of positive reinforcement as previously tested. No statistically significant differences between the groups were found.

**Results:**

Overall, the findings suggest that both primary reinforcement alone as well as a verbal or clicker secondary reinforcer can be used successfully in training a dog to perform a novel behavior, but that no positive reinforcement method demonstrated significantly greater efficacy than any other.

## Introduction

In positive reinforcement training, a trainer increases the frequency of a target behavior by regularly following it with a primary reinforcer (a stimulus which has reinforcing efficacy without the need for any other behavioral manipulation). A trainer may also present a secondary reinforcer—a stimulus which is initially neutral but which acquires the ability to increase the likelihood of a response by being repeatedly paired with a primary reinforcer (Domjan, 2005). In principle, a secondary reinforcer can be anything the animal perceives—such as a light, a sound, a movement, or a smell. Common secondary reinforcers in dog training are a spoken word, a whistle, or the sound from a clicker device (Burch & Bailey, 1999; Schaefer, 1999; Skinner, 1951).

Despite the prominence of positive reinforcement-based training methods in the professional dog training community (Blackwell et al., 2008; Hiby, Rooney & Bradshaw, 2004), recommendations of how, when, and what method of positive reinforcement should be used are inconsistent (see Browne et al. (2017), for a review of the general content in best-selling dog training books). Indeed, more generally, the research reveals mixed results as to the best approach to train animals. Studies in other species have shown that, as compared to the use of both a primary and secondary reinforcer, the use of a primary reinforcer alone when establishing a new behavior is more effective in cats (Willson et al., 2017), but less effective in goats (Langbein et al., 2007), and equally effective in horses (McCall & Burgin, 2002).

A widely promulgated form of secondary reinforcement in dog training is the use of a handheld metal noisemaker known as a “clicker” that is first paired with food, after which the “click” can be used to audibly signal the performance of a desired behavior in the animal (Pryor, 1999). There is somewhat more consistency in the training literature with regards to the efficacy of clicker training for behavior acquisition—namely, that it is often no better than other secondary reinforcers or the use of primary reinforcement alone. In some of the earliest studies to compare clicker training to primary reinforcement alone in domesticated animals, similar protocols were used to shape horses (Williams et al., 2004) and dogs (Smith & Davis, 2008) to perform the novel behavior of touching their nose to a cone with either primary reinforcement alone or the sound of the clicker followed by primary reinforcement. The behavior was then placed under extinction, with secondary reinforcement administered to half of the clicker-trained horses and all of the clicker-trained dogs. Neither study found a difference in the number of trials the animals required to reach training criterion across training conditions, and while Williams et al. (2004) found that neither condition nor administration of secondary reinforcement during extinction influenced the total number of trials required to reach extinction, Smith & Davis (2008) found that dogs trained with the clicker required significantly more time and trials to extinguish the behavior when primary reinforcement was discontinued. Although the continued presence of a secondary reinforcer is expected to slow behavioral extinction due to a less pronounced change in the stimulus configuration from training to extinction (Pearce, 2008; Williams, 1994), Smith & Davis (2008) suggested that the difference between their findings with dogs and Williams et al. (2004) results with horses could be a true species difference in behavioral processes. Additionally, factors such as heightened arousal to the sound of the mechanical clicker, frustration, or the inability to discriminate between testing and extinction conditions (Kelleher & Gollub, 1962; Williams, 1994) could also play a role in increased resistance to extinction for clicker-trained dogs but not horses. Previous studies have compared the relative efficacy of using primary reinforcement alone to the sound of the clicker followed by primary reinforcement when teaching a new behavior, but little is known about the relative efficacy of the clicker compared to other forms of secondary reinforcement.

Two prior studies compared the efficacy of a clicker to a verbal secondary reinforcer in training a novel behavior in dogs and piglets. Thorn et al. (2006) measured the latency to sit upon approach for shelter dogs trained with a clicker compared to a verbal secondary reinforcer. They found no differences in any dependent measures between conditions on the first day of training. On the second day of training, dogs trained with a clicker had a significantly greater latency to sit during their first trial compared to their last trial on the first day and had a significantly greater mean latency to sit on the second day overall. This indicates that a clicker or a verbal secondary reinforcer may be equally efficacious for initially training a behavior, but that clicker-trained dogs have a lower retention of the behavior across days. In a recent study with piglets, Paredes-Ramos et al. (2020) used a clicker or a verbal secondary reinforcer to first teach a “fetch” behavior, and then had the piglets fetch a novel object in a discrimination task. They found that clicker-trained piglets acquired the fetch behavior in significantly fewer trials than the verbally-reinforced piglets, but that verbally-reinforced piglets made significantly more correct choices on the discrimination task. However, both studies omitted a primary reinforcer alone condition, thereby making assessment of putative reinforcing effects difficult.

To date, two studies have investigated the effect of shaping a novel behavior using primary reinforcement alone or in association with a spoken word or a clicker. Chiandetti et al. (2016) trained dogs to open a bread box using a clicker paired with food, the spoken word “bravo” paired with food, or food alone, and then tested whether the dogs could generalize that behavior to a new apparatus. They found that dogs in all three conditions were able to learn and generalize the behavior to an equivalent degree. Using these same three reinforcement types, with the spoken word “next” instead of “bravo” as the verbal reinforcer, Dorey, Blandina & Udell (2020) shaped a stay behavior in one set of puppies, and a wave behavior in second set of puppies. They found that puppies in the stay behavior group trained with food alone progressed significantly further in shaping approximations than those trained with the clicker, with verbally-reinforced puppies reaching an intermediate shaping approximation. When teaching the wave behavior, Dorey, Blandina & Udell (2020) saw no differences across the three conditions.

Several procedural factors may contribute to divergent findings from these studies of primary and putative secondary reinforcement in dogs, and differences in the design of prior studies make it difficult to reconcile their disparate results. For example, Marshall-Pescini et al. (2008) have shown that dogs with more training experience performed significantly better when learning a new task than dogs without prior training. In the studies that have investigated the use of a clicker during dog training, Smith & Davis (2008) and Chiandetti et al. (2016) enrolled dogs 6 months to 12 years old and 8 months to 13 years, respectively. These dogs had prior training experience, but no prior exposure to a clicker. Thorn et al. (2006) utilized dogs over a year old of unknown ownership, and consequently unknown training histories. As dogs in shelters typically have unknown training histories, the best way to reduce the likelihood of such experience influencing dogs’ performance is to test younger dogs. Dorey, Blandina & Udell (2020) addressed this concern by using shelter puppies 8–24 weeks old in their experiments. Additionally, it is possible that the frequency and duration of training sessions may impact behavioral acquisition. Demant et al. (2011) found that dogs trained to sit and stay in a basket once or twice weekly reached a significantly higher acquisition level than those trained daily, as did those trained with only one session as opposed to three consecutive sessions per day. Thorn et al. (2006) conducted testing over 2 days each separated by 2 days. Smith & Davis (2008) used from 2 to 6 days of consecutive training while Chiandetti et al. (2016) tested over “several” days. And while the number of overall trials was controlled by Demant et al. (2011), Thorn et al. (2006), and Dorey, Blandina & Udell (2020), dogs in both Smith & Davis (2008) and Chiandetti et al. (2016) were offered unlimited trials to reach session criteria. Differences in training method efficacy may also relate to the type or complexity of the behavior being trained. Fugazza & Miklósi (2015) found that significantly more dogs learned to open a sliding door when trained with the “Do as I do” method than those shaped with a clicker, but that training method did not influence their ability to learn to jump in the air. Studies that taught a relatively simple behavior like sit and stay found differences in behavior acquisition (Dorey, Blandina & Udell, 2020) and retention (Thorn et al., 2006), whereas no difference in training method efficacy was seen for teaching more complex behaviors like targeting (Smith & Davis, 2008), waving (Dorey, Blandina & Udell, 2020), and object manipulation (Chiandetti et al., 2016).

Given that prior studies varied along multiple procedural dimensions that could have affected acquisition of a novel behavior, we saw the need for a cohesive set of experiments that utilized participants of a similar age, with similar training histories, and that received the same amount of time to complete the testing protocol. By utilizing a single-session design and testing puppies naïve to training, we believe that any indication of greater performance by clicker-trained dogs could be more clearly related to the relationship between the reinforcement method and the behavior being trained.

The aim of the present study was to clarify whether differences exist in the rate of acquisition and (equivalently) terminal level reached in a fixed number of trials when establishing novel behaviors in dogs with one of three commonly used positive reinforcement methods: Primary Alone, Verbal Secondary, or Clicker Secondary. In the first experiment, each dog was taught to sit for increasing periods of time using one of the three reinforcement methods, and the differences in the greatest durations of sit achieved in a fixed number of trials were compared between groups to determine whether one condition resulted in the dogs learning the behavior more rapidly and sitting for a longer duration than the others. In the second experiment, dogs were first shaped to touch a cone next to the trainer, and then required to target the cone at increasing distances. Both the dogs’ progress through the shaping approximations and the greatest distance from the cone that the dogs achieved in a fixed number of trials were compared between groups to determine which condition facilitated the highest level of behavioral acquisition. In the final experiment, dogs were shaped to touch an array of blocks, and then progressed through levels of increasing specificity wherein only targeting specific blocks was reinforced. The shaping approximation that was achieved, the amount of time it took to complete each approximation, and the number of attempts made during those shaping levels were compared between groups to determine which condition resulted in the greatest degree of behavioral specificity in a fixed number of trials.

## General design

Testing was conducted inside a conference room at the Arizona Humane Society (AHS: Phoenix, AZ, USA). Metal fencing was used to enclose the testing area in each experiment, with enclosure size varied across experiments. Participants were puppies from AHS, 8–22 weeks old, that came from the shelter’s intake and adoption areas. This age range was chosen so that dogs were physically capable of completing the tasks but unlikely to have experienced prior training. Dogs had to be free of illness, injury, and anesthetics to participate. Per shelter protocol, all dogs received their regular diet in the morning and evening while the studies were being conducted and were not tested immediately after meal consumption. No dog participated in more than one experiment. All procedures in this study were conducted with approval from the Arizona State University Institutional Animal Care and Use Committee (16-1462R RFC2; 19-1668R).

RG served as the trainer throughout the experiment^1^ 1RG had seven years of experience with shelter dogs and an undergraduate degree in biology, but no explicit credentials of training., and all individuals handling the dogs were female. After an opportunity to urinate and defecate, each dog was placed in the testing area and given 4 min to explore. The trainer only interacted with the dog if approached. Next, the trainer stood and placed the clicker on her left middle finger and two treat bags along her shirt collar (Experiments 1 and 2) or a treat bag along her waistband on her lower back (Experiment 3). One bag contained the primary reinforcer (pieces of hot dog), while the other (in Experiments 1 and 2) contained pieces of Pup-peroni® Original Beef Flavor (Big Heart Pet, Inc.). The latter treats were tossed into Zone 2 after a dog emitted a behavior in Zone 1 in order to bring the dog back into Zone 2 for the start of each trial. This was done to get the dog out of the reinforced behavior for the experiment (sit or touch) without manhandling it. It also allowed the dog to start every trial in Zone 2, thereby enabling us to assess its motivation to engage in the next trial, which would otherwise have been difficult to do if the dog remained in Zone 1 for the duration of the test.

To ensure the dog did not have prior experience of sitting (Experiment 1) or targeting (Experiments 2 and 3) when prompted, the trainer approached it, said “sit,” (Experiment 1) or presented her empty palm (Experiments 2 and 3), and waited for a response three times. If the dog sat or touched three times, it was excluded from participation. If it sat or touched once or twice, the trainer allowed 30 s to elapse before the dog was again prompted three times. If the dog sat or touched for two or all three prompts, it was removed from the experiment. Dogs that sat or touched only once remained in the experiment.

Dogs were randomly assigned to one of three groups: Primary Alone, Verbal Secondary, or Clicker Secondary. Dogs in the two secondary reinforcement groups received a piece of hot dog paired with the verbal sound “chee” or with the click from a clicker as their reinforcement. Dogs experienced the pairing of the primary reinforcer with the secondary reinforcer 20 times in succession, with each pairing beginning immediately after consumption of the previous piece of food, which is consistent with or exceeds the number of pairings used by Thorn et al. (2006), Smith & Davis (2008), Chiandetti et al. (2016), and Dorey, Blandina & Udell (2020). To control for the amount of food received prior to the start of training, and any association thereby formed between the experimenter and food, dogs in the primary alone group also received 20 primary reinforcers with the trainer silently thinking “chee” before each presentation of the primary reinforcer to mimic the approximate duration it took to deliver the secondary reinforcers in the other groups. Dogs that did not consume the primary reinforcer were removed from the experiment.

## Experiment 1

In the first experiment, we tested whether there were any differences in the maximum duration a dog could be trained to sit for primary reinforcement only, secondary reinforcement from a verbal stimulus, and secondary reinforcement from a clicker in a fixed number of trials.

### Setting and subjects

**Figure 1:**
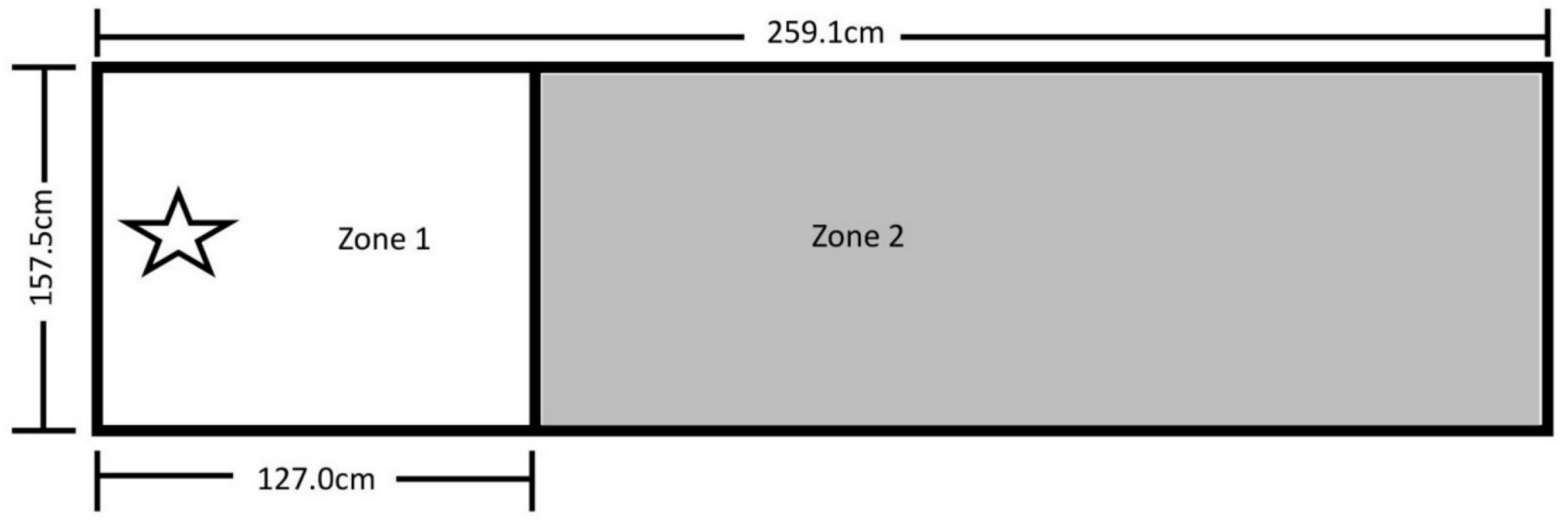
Testing layout in Experiment 1. The 157.5 cm by 259.1 cm enclosed space making up the testing area. A line of tape separating Zone 1 (where sitting was reinforced) from Zone 2 (no reinforced behavior) was placed 127.0 cm from the far end of Zone 1. The star marks where the trainer stood during testing and where reinforcement delivery occurred (the RDS). A research assistant was present on the far right end of Zone 2.

The testing area for Experiment 1 was 157.5 cm by 259.1 cm and was comprised of Zone 1 on the left, where the dog was reinforced for sitting, and Zone 2 on the right, where it was not reinforced for its behavior (Fig. 1). A video camera was set up to record all testing within the fenced area.

Of the 110 dogs that participated in this study (Table S1), seven sat on command, implying prior training; another 13 did not sit within 25 min of initiating training; and one was later revealed to have been ill during testing, thus excluding it from analysis (see “Procedure” below). The final sample size consisted of 29 dogs in the primary alone group and 30 each in both the verbal and clicker secondary groups. A power sensitivity analysis was conducted using G*Power (Faul et al., 2007) to assess what magnitude of effect could be reasonably detected with this final sample size. These calculations showed that a sample size of 89 dogs has 80% power to detect a medium effect size of *f* = 0.33 for group differences, controlling for covariates of age, sex, and weight.

### Procedure

After the pairing of secondary reinforcers was complete, the trainer stood at the far-left end of Zone 1 on the Reinforcement Delivery Spot (RDS—see Fig. 1). Each dog was given 25 min to enter Zone 1 and sit for one second. If a dog did not perform the behavior within this time, it was excluded from further participation. If the dog did sit within Zone 1 for 1 s, it received its assigned reinforcement, and a Pup-peroni treat was thrown across the line into Zone 2 to encourage the dog to leave Zone 1 before initiating a new trial. If the dog did not follow the treat into Zone 2, the trainer walked to where the treat landed and verbally encouraged the dog to retrieve it, and then returned to the RDS once it left Zone 1 to consume the treat.

**Figure 2:**
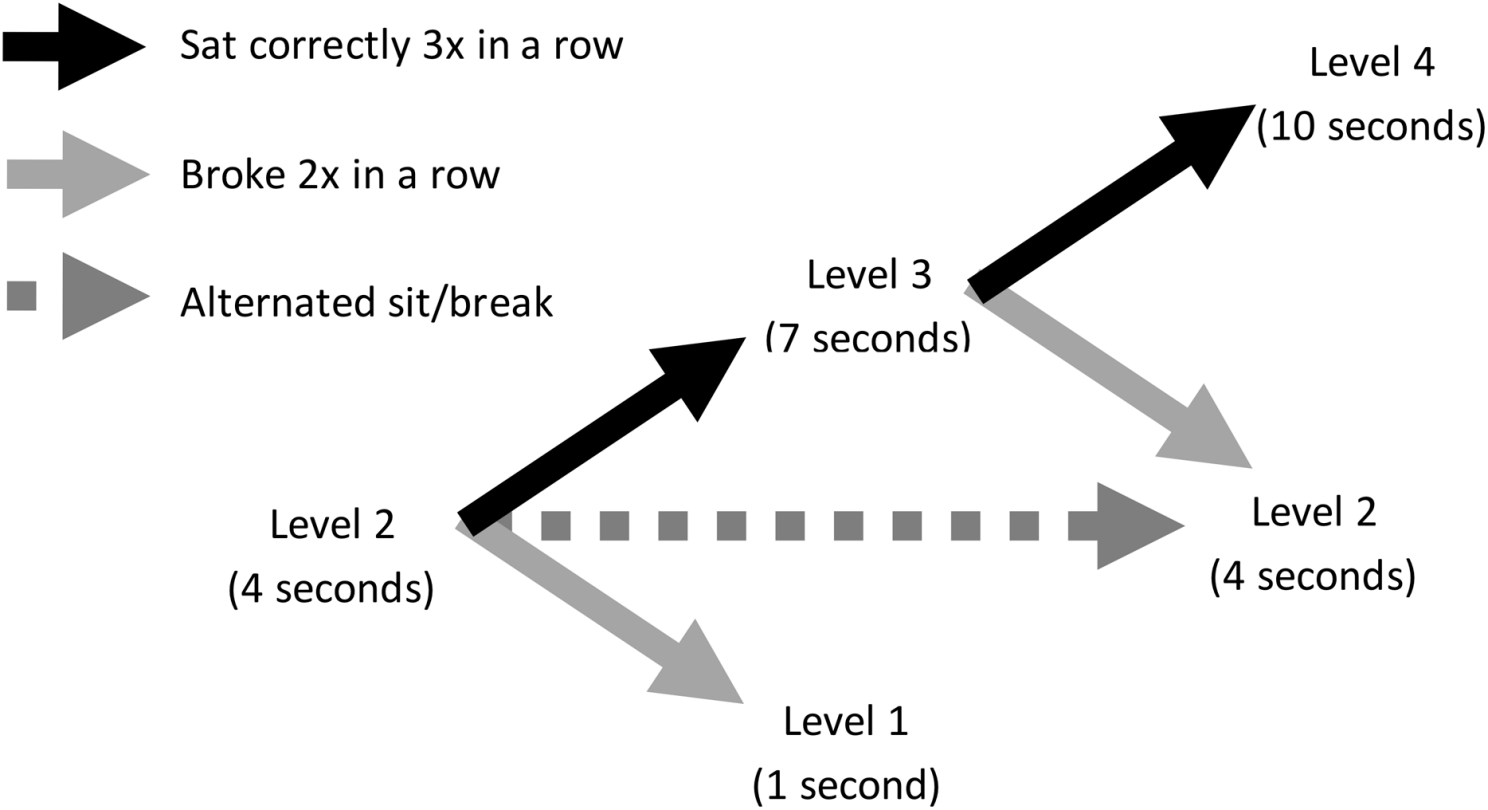
Adaptive schedule of reinforcement in Experiment 1. Illustration shows an example dog at Level 2, where it was required to sit for 4 s to obtain reinforcement. If it sat for 4 s three times in a row, it would advance to Level 3, where it was expected to sit for 7 s. If, however, at Level 2 the dog did not sit for a full 4 s twice in a row, it would drop down to Level 1, where it was expected to sit for 1 s. If the dog alternated sitting for 4 s and sitting for less than 4 s, it remained at Level 2.

All dogs were initially required to sit for 1 s to receive reinforcement, and the sitting criterion increased in 3 s intervals as they performed a sit for the required duration three times in succession. Dogs received reinforcement at the end of each sit that met criterion. If the dog did not sit for the entire duration, it did not receive reinforcement, but still received a treat thrown into Zone 2 to start the next trial. If the dog sat for less than the current duration criterion twice in a row, the sit duration was reduced by 3 s. If the dog alternated performing criterion compliant and noncompliant sits, it remained at its current criterion level until it successfully sat correctly three times in succession or failed to reach criterion twice in succession. Figure 2 diagrams this adaptive schedule of reinforcement. For every 15 s that the dog spent in Zone 2, the trainer would call to it by saying “puppy ba ba ba” and made a kissing sound to encourage it to return to Zone 1. Each sit constituted one trial, and each dog could perform a maximum of 50 trials. Data were retained and analyzed for dogs that completed at least one trial. A dog’s testing ended either when it did not perform another sit within Zone 1 within 2 min from its last reinforced sit, or once it reached 50 trials.

## Results and discussion

**Figure 3:**
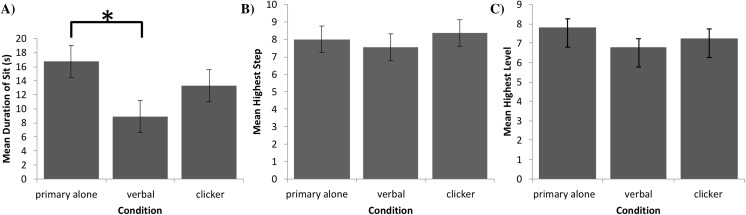
Task performance in Experiments 1, 2, and 3. (A) Mean duration of a sit (with error bars depicting standard error of the mean) in seconds for each group of dogs in Experiment 1. Bracket with asterisk between groups indicates a significant difference in means on post-hoc pairwise comparisons (*p* < 0.05). (B) Mean highest completed step (with error bars depicting standard error of the mean) for each group of dogs in Experiment 2. (C) Mean highest completed level (with error bars depicting standard errors of the mean) for each group of dogs in Experiment 3.

The final sample size was *n* = 29 in the primary alone group and *n* = 30 in each of the other two groups. After verifying the assumptions of normality and homogeneity of variances of residuals with a Shapiro–Wilk test (*W*(89) = 0.97, *p* = 0.054), and a Levene’s test (*F*(2, 86) = 2.94, *p* = 0.06), and confirming that no standard deviation of one group exceeded twice the standard deviation of any other group (SD_primary_ = 11.7 ; SD_verbal_ = 8.8 ; SD_clicker_ = 11.1), we performed a one-factor ANCOVA in SPSS (Version 25) International Business Machines Corp. (2017) with age, sex, and weight as covariates to compare the influence of group on the maximum duration of the dogs’ sitting behavior. Figure 3A provides the means and their standard errors for each group. The ANCOVA revealed a statistically significant difference in durations of sit between groups (*F*(2, 83) = 3.95, *p* = 0.02), with no covariate reaching statistical significance: *p* = 0.57 for age, *p* = 0.85 for sex, and *p* = 0.28 for weight. Furthermore, Tukey post-hoc testing revealed a significant difference between the verbal secondary and primary alone groups (*p* = 0.01, *d* = 0.76, 95% CI [0.23 to 1.28]), while the differences between the clicker secondary and primary alone groups (*p* = 0.22, *d* = 0.33, 95% CI [−0.19 to 0.84]) and clicker and verbal secondary groups (*p* = 0.10, *d* = 0.43, 95% CI [−0.08 to 0.94]) were not statistically significant^2^ 2There are multiple ways of calculating Cohen’s *d* in designs with covariates. We used adjusted means and the square root of the mean square error from an ANOVA, rather than an ANCOVA, model in all of our calculations, based on methodological recommendations (Maxwell, Delaney & Kelley, 2018; Olejnik & Algina, 2000). Tukey’s multiple comparison procedure appropriately controls the family-wise Type I error rate at 0.05 (Maxwell, Delaney & Kelley, 2018)..

To test whether the superior performance of the primary alone group was due to the lack of an intervening delay imposed by the delivery of the secondary reinforcer, we randomly selected 12 out of 30 dogs in each group and analyzed the first, middle, and last 2-min segments of their testing sessions to assess the delay to reinforcement they experienced using CowLog (Version 3.0.2; Pastell, 2016). Coders blind to the hypotheses and purpose of the experiment coded the delays to reinforcement of these dogs. The average delays to reinforcement per condition for the clicker and verbal secondary groups were 2.5 s (SD = 0.7) and 2.6 s (SD = 0.7) respectively, and that of the primary alone group was 3.1 s (SD = 1.5). Thus, the greater efficacy of dogs in the primary alone group cannot be attributed to a shorter delay to reinforcement than the secondary reinforcement groups.

It is possible that dogs in the verbal secondary group performed significantly worse than those in the primary alone group because they had been habituated to the human voice. Although we used the uncommon and likely unfamiliar sound “chee” as our verbal secondary reinforcer to try to control for words that the dogs already had experience with, it is possible that the constant exposure that dogs have to the human voice had desensitized them such that any spoken word could not prove to be a strongly effective secondary reinforcer (Pearce, 2008).

One possible explanation for the failure to detect a benefit of the clicker in this experiment may be that the primary reinforcer was delivered promptly. Pryor (2009), in arguing for the effectiveness of clickers, gave an example where a click sound was used to signal reinforcement to the animal when it performed a behavior at a distance from the trainer. Under conditions where primary reinforcement cannot be provided quickly, secondary reinforcement can provide feedback to an animal that it has behaved correctly and will receive primary reinforcement upon returning to the trainer. Experiment 2 sought to investigate whether secondary reinforcement could have an impact on the performance of a behavior at a greater distance from the trainer.

## Experiment 2

To test whether secondary reinforcement could have a greater impact on the acquisition of novel behavior at a greater distance from the trainer, in Experiment 2 we trained dogs under the same three positive reinforcement methods from Experiment 1, but shaped them to touch a cone at increasing distances from the trainer. This targeting behavior preceded the distance component of testing.

### Setting and subjects

**Figure 4:**
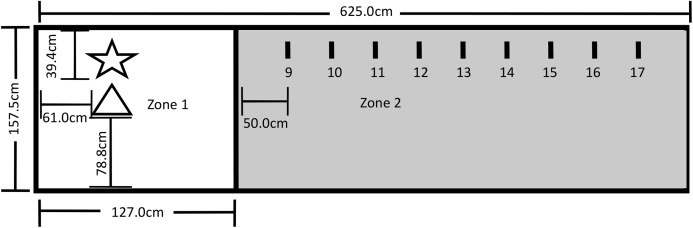
Testing layout in Experiment 2. The star represents where the trainer stood during testing and where primary reinforcement delivery by the trainer occurred (PRDS). The triangle represents the touch spot where all reinforced behavior occurred and where primary reinforcement was delivered (TS). The short lines represent the upper step markers (USM) 9–17 that marked where the trainer stood for Steps 9–17. The USM were placed in line with the PRDS and were spaced 50 cm apart from one another, with USM 9 being 50 cm away from the line. A research assistant was present on the far end of Zone 2.

The testing area for Experiment 2 was a 625.0 cm by 157.5 cm area comprising Zone 1, which included the Touch Spot (TS) and the Primary Reinforcer Delivery Spot (PRDS), and Zone 2, which included the Upper Step Markers (USM). The layout of the testing area is shown in Fig. 4. All reinforced behavior and delivery of primary reinforcement occurred at the TS. The trainer stood on the PRDS when delivering the primary reinforcer over the TS, and both knelt and stood on the PRDS during Steps 1–7. A research assistant sat outside the fencing at the far-right end of Zone 2 during training, and a video camera was set up to record all testing within the fenced area.

Of the 84 dogs that participated in this study (Table S2), nine did not touch the trainer’s hand with their nose within 25 min of initiating training (see “Procedure” below) and were excluded from participating further. Twenty-five dogs in each group touched the trainer’s hand with their nose at least once, and their data were analyzed. Power calculations to determine the sensitivity of effect size detection for the final sample size were conducted using G*Power (Faul et al., 2007). The sensitivity analysis showed that a sample size of 75 dogs has 80% power to detect a medium effect size of *f* = 0.37 for group differences, controlling for covariates of age, sex, and weight.

### Procedure

After the pairing of secondary reinforcers was complete, the trainer walked to the PRDS and knelt holding out a primary reinforcer in her right palm over the TS. The dog had 25 min to approach and nose-touch her palm. If the dog did not do this within the time allotted, it was removed from the study. If the dog touched her palm with its nose, it received its assigned reinforcement, and a treat was thrown into Zone 2 to encourage it to leave the trainer’s side. If the dog did not follow the treat into Zone 2, the trainer tossed up to two more treats, after which point the research assistant would call it toward the far-right side of Zone 2.

To measure the effectiveness of the three reinforcement methods, each dog was first shaped to perform the nose-targeting behavior, and then to perform this behavior at increasing distances from the trainer. Table 1 shows all successive approximations used in the shaping procedure. Dogs received their designated reinforcement when they nosed the object as required by the current step. All dogs were required to complete Steps 1–7 before a distance component was introduced to the testing. At Step 8, the experimenter pivoted on the PRDS and took one step backward from the PRDS to the line. For Steps 8–17, the dog was tasked with nose-touching the tip of the cone that was placed on the TS just as it had done in Steps 6 and 7.

**Table 1:** Testing Steps of Experiment 2. List of each testing step and corresponding reinforced behavior, as well as the trainer’s location and posture at each step.

Step	Reinforced behavior	Trainer location, stature
1	Touch trainer’s palm containing food	PRDS (0 cm from cone), kneeling
2	Touch trainer’s empty palm	PRDS (0 cm from cone), kneeling
3	Touch ball in trainer’s hand	PRDS (0 cm from cone), kneeling
4	Touch ball affixed to cone held in trainer’s hand	PRDS (0 cm from cone), kneeling
5	Touch ball affixed to the cone placed on TS	PRDS (0 cm from cone), kneeling
6	Touch cone placed on TS	PRDS (0 cm from cone), kneeling
7	Touch cone placed on TS	PRDS (0 cm from cone), standing
8	Touch cone placed on TS	Line (127 cm from cone), pivot, standing
9	Touch cone placed on TS	USM 9 (177 cm from cone), standing
10	Touch cone placed on TS	USM 10 (227 cm from cone), standing
11	Touch cone placed on TS	USM 11 (277 cm from cone), standing
12	Touch cone placed on TS	USM 12 (327 cm from cone), standing
13	Touch cone placed on TS	USM 13 (377 cm from cone), standing
14	Touch cone placed on TS	USM 14 (427 cm from cone), standing
15	Touch cone placed on TS	USM 15 (477 cm from cone), standing
16	Touch cone placed on TS	USM 16 (527 cm from cone), standing
17	Touch cone placed on TS	USM 17 (577 cm from cone), standing

Each nose-touch or failure to touch the cone or experimenter’s palm constituted one trial, and each dog was permitted to perform a maximum of fifty trials and a maximum of 17 steps. Data were retained and analyzed for dogs that completed at least one trial. For every 15 s that the dog spent in Zone 2, the trainer called to it by saying “puppy ba ba ba” and making a kissing sound to encourage it to return to Zone 1. If the dog did not enter Zone 1 within 2 min of its last nose-touch, testing ended. If the dog remained inside Zone 1 for 2 min without emitting a nose-touch, it was considered a failed trial and a treat was thrown into Zone 2 to initiate the next trial. If this occurred three times successively, testing ended. If the dog crossed the line with at least one forepaw into Zone 1, but did not nose-touch, it was considered a failed trial. Once a dog reached Step 8, it was not permitted to return to earlier shaping steps after two failed attempts at Step 8, and testing was ended.

## Results and discussion

Data from 25 dogs in each group were analyzed. After verifying the assumptions of normality and homogeneity of variances of residuals with the Shapiro–Wilk test (*W*(75) = 0.971, *p* = 0.08) and Levene’s test (*F*(2, 72) = 1.14, *p* = 0.33), and confirming that no standard deviation of one group exceeded twice the standard deviation of any other group (SD_primary_ = 3.4 ; SD_verbal_ = 3.5 ; SD_clicker_ = 4.4), we performed a one-factor ANCOVA in SPSS (Version 25) International Business Machines Corp. (2017) with age, sex, and weight as covariates to compare the influence of group on completed levels. Figure 3B provides the means and standard error of the means for each group. The ANCOVA indicated that the difference in number of steps completed between groups was not statistically significant, *F*(2, 69) = 1.10, *p* = 0.37, with no covariate reaching statistical significance, *p* = 0.57 for age, *p* = 0.71 for sex, and *p* = 0.07 for weight. Effect sizes for the three pairwise differences in the number of steps completed were *d* = 0.12 (95% CI [−0.43 to 0.68]), *d* = 0.09 (95% CI [−0.46 to 0.65]), and *d* = 0.22 (95% CI [−0.34 to 0.78]), for comparing verbal secondary to primary alone, clicker secondary to primary alone, and clicker secondary to verbal secondary, respectively.

Although we should be careful when making inferences regarding non-significant results, it should be noted that, on average, dogs in the clicker secondary group completed an additional shaping step than those in the verbal secondary group, with the primary alone group achieving an intermediate number of steps. Moreover, the greatest proportion of verbal secondary-group dogs and the smallest proportion of clicker secondary-group dogs dropped out at or before Step 7—the final shaping step before distance was added to the task, with the primary alone group dropping out at an intermediate number of steps.

In order to test for potential differences in delay to reinforcement between the three groups, we randomly selected 12 out of 25 dogs in the primary alone and clicker secondary groups and 10 out of 25 in the verbal secondary group and had coders blind to the study aims and methods analyze the first, middle, and last 2-min segments of their testing videos for delays to primary reinforcement by using BORIS (Version 5.1.0; Friard & Gamba, 2016). Average delays to reinforcement per condition for the primary alone, verbal secondary and clicker secondary groups were 3.0 s (SD = 1.4), 2.5 s (SD = 1.4) and 4.0 s (SD = 2.0) respectively. While it is possible that dogs in the clicker secondary group were negatively impacted by the greater delay to reinforcement they experienced compared to the other reinforcement groups, it should be noted that, because, on average they completed more steps in the distance phase of training than dogs in either of the other two groups, they necessarily waited longer as the trainer walked the additional distance to deliver primary reinforcement to them.

In this experiment we did not have enough statistical power to analyze differences between the groups in the shaping of the targeting behavior separately from the distance-related behavior. Future studies with a larger sample size may be able to separately analyze performance on the initial shaping steps and the subsequent steps where the behavior was performed at a distance from the experimenter. Overall there were no statistically significant differences between the groups.

Our aim thus far has been to find the circumstances in which a clicker may result in acquisition of a novel behavior to a higher level than other readily available reinforcement methods. Experiment 1 found no advantage to secondary reinforcement in training a simple sit behavior. Experiment 2 tested the hypothesis that clickers aid in training a behavior performed at a distance from the trainer (Pryor, 2009) and found no one reinforcement group better sustained a targeting behavior over increasing distances. Feng, Howell & Bennett (2018) reported that owners and trainers believe clickers to be more beneficial when teaching discrete behaviors, such as targeting, rather than less specific ones, such as coming when called—a belief also consistent with Pryor (2009). Consequently, in Experiment 3 we test whether the clicker secondary reinforcer allows dogs to reach a higher criterion of behavioral acquisition in a fixed number of trials when the specificity of the behavior is the focus of training.

## Experiment 3

In Experiment 3, dogs were tasked with learning to emit a nose-targeting behavior on an array of alternating yellow and blue wooden blocks. Initially nose-targeting any block in the array was reinforced, but in subsequent phases of training only dogs’ contact with specific blocks was reinforced. Table 2 gives the criteria for progression through the testing levels. Not only did Pryor (2009) propose that clicker training is beneficial due to the speed and precision with which it could be employed, but she also stated that the clicker is intrinsically reinforcing for dogs and aids in keeping them engaged with a task because it activates the SEEKING circuit. Panksepp’s (2010) theory of a SEEKING circuit states that “SEEKING coaxes animals to acquire resources needed for survival. It promotes learning by mediating anticipatory eagerness, partly by coding predictive relationships between events” (p.538). Consequently, Panksepp’s (2010) theory of a SEEKING circuit predicts that dogs trained using a clicker may make more contact with the blocks in this experiment because engagement of the SEEKING circuit increases the reinforcing qualities of the apparatus. Thus, we sought to determine whether dogs in the clicker secondary group attained higher levels of acquisition of this new behavior when compared to those in the primary alone or verbal secondary groups, and whether they attained different rates of responding at each level.

**Table 2:** Shaping and Testing Levels of Experiment 3. Outline of the shaping and testing phases, as well as all possible levels and their corresponding blocks eligible for reinforcement when touched.

Level	Blocks eligible for reinforcement
Training/Shaping	One randomized block is eligible for reinforcement; all blocks are eligible once
1	All blocks are eligible for reinforcement
2	Blocks 2, 3, and 4 are eligible for reinforcement
3	A randomized pair of blocks (either 1 and 2 or 3 and 4) are eligible for reinforcement
4	A randomized pair of blocks (either 1 and 2 or 3 and 4) are eligible for reinforcement; not the same pair as level 3
5	Blocks 1, 3, and 5 are eligible for reinforcement
6	Blocks 2 and 4 are eligible for reinforcement
7	One randomized block is eligible for reinforcement
8	One randomized block is eligible for reinforcement; not the same block as Level 7
9	One randomized block is eligible for reinforcement; not the same block as Levels 7 or 8
10	One randomized block is eligible for reinforcement; not the same block as Levels 7–9
11	One randomized block is eligible for reinforcement; not the same block as Levels 7–10

### Setting and subjects

**Figure 5:**
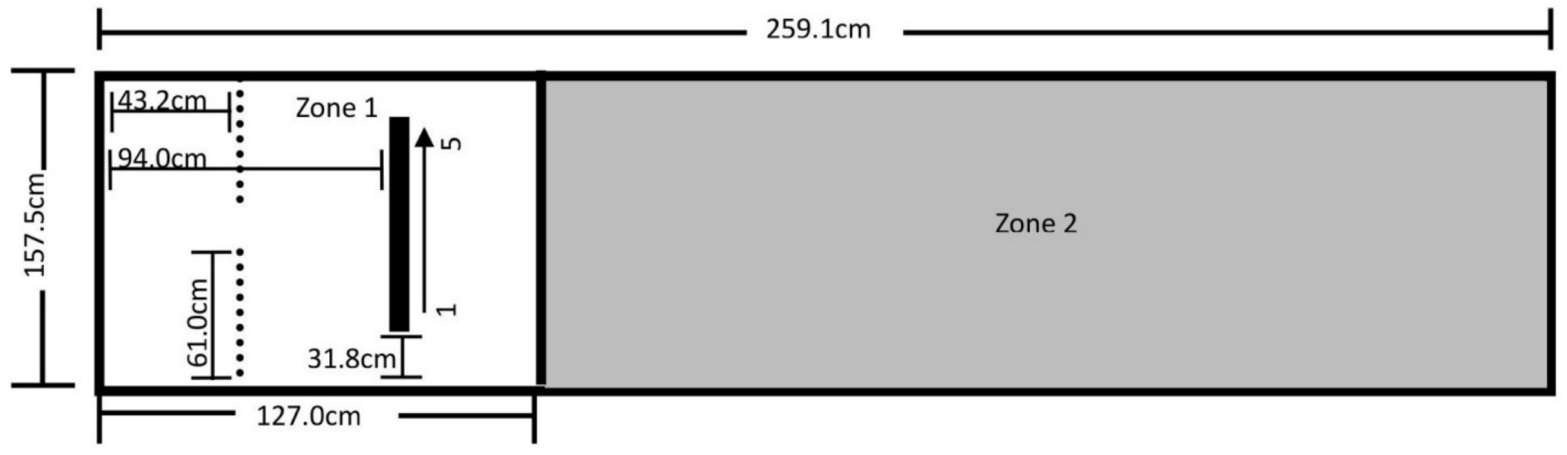
Testing layout in Experiment 3. The tape line dividing the zones sits 127.0 cm from the far left end of Zone 1. The thin dotted lines represent the Reinforcement Barrier (RB), between which the trainer knelt during testing and behind which sat the primary reinforcement and resetting treats. The black bar represents the Block Line (BL) where all reinforced behavior occurred and where primary reinforcement was delivered, and the arrow from 1 to 5 indicates the numbered direction of the blocks.

The testing area for Experiment 3 was a 157.5 cm by 259.1 cm area comprising the Reinforcement Barrier (RB: two 61.0 cm by 91.4 cm tri-fold cardboard barriers supported by metal table easels), the Block Line (BL), and Zones 1 and 2, as shown in Fig. 5. The trainer knelt in the 35.6 cm space between the barriers during testing. The BL was comprised of a 94.0 cm by 2.4 cm by 1.3 cm metal bar secured to the floor with Velcro tape, and had five 3.8 cm by 6.4 cm by 9.8 cm magnetic painted wood blocks evenly spaced 18.7 cm apart along the length of the bar. All reinforced behavior and delivery of primary reinforcement occurred along the BL. A research assistant sat outside the fencing at the far-right end of Zone 2 during training, and a video camera was set up to record all testing within the fenced area.

One hundred and twelve dogs participated in the third experiment (Table S3). Of these, one was excluded because it touched an empty outstretched palm, implying prior training; another was excluded for vomiting during testing; and one would not eat the primary reinforcer. Nineteen dogs were excluded for not touching all five blocks within 25 min of testing, thus excluding a total of 22 dogs from the analysis. A power sensitivity analysis was conducted using G*Power (Faul et al., 2007), which showed that the final sample size of 90 dogs has 80% power to detect a medium effect size of *f* = 0.25 for the focal between-group effect, controlling for the covariates of age, sex, and weight.

### Procedure

After the pairing of secondary reinforcers was complete, the trainer led the dog to a research assistant, who faced it away from the testing enclosure while the trainer assembled the testing apparatus. The primary reinforcer and the treat used to move dogs to Zone 2 after completed trials were placed in bowls behind the right RB (from the trainer’s perspective when facing the dog), and a cell phone running a timer was placed behind the left RB. The trainer placed each of the five blocks on the BL, then knelt between the RBs and placed a primary reinforcer on a predetermined, randomly-assigned block. The research assistant returned the dog to the testing enclosure, and it was given 25 min to enter Zone 1 and touch the block with the primary reinforcer on top of it. If the dog did not perform this behavior within the 25 min, it was removed from the experiment. If the dog nose-touched the block with the primary reinforcer on it, it received its assigned reinforcement, and a treat was thrown into Zone 2 to encourage the dog to leave Zone 1 before returning for the next trial. While the dog retrieved this treat, the trainer placed a primary reinforcer on a different predetermined randomly-assigned block, and the process was repeated until all five blocks had been nose-touched once, thus completing the shaping phase.

**Figure 6:**
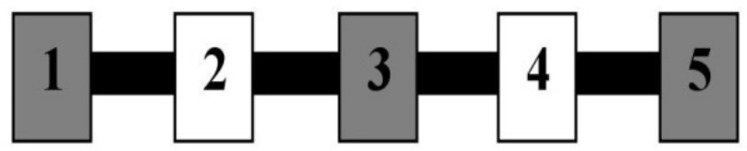
Testing apparatus in Experiment 3. Blocks are numbered from the trainer’s left to right, beginning at Block 1. Consecutive blocks alternated colors, with a 3 blue/2 yellow and 3 yellow/2 blue arrangement assigned to equal numbers of dogs in each condition. Blocks were evenly spaced 18.7 cm apart.

Once the dog had the experience of touching each block individually, it was then required to perform this behavior on increasingly specific groupings of blocks. The testing phase consisted of 11 levels, each comprising a different arrangement of blocks that were eligible for reinforcement if nose-touched. A dog advanced to the next level only when it touched a correct block with its nose four times successively. Dogs were permitted an unlimited number of attempts to nose-touch the blocks until they touched one eligible for reinforcement, at which time primary reinforcement was delivered directly above the block that it touched. Table 2 and Fig. 6 show details of the progressively more specific testing phases.

Each dog had 30 min to complete the shaping and testing phases. Data were retained and analyzed for dogs that completed the shaping phase and were reinforced for at least one nose-touch at Level 1. If a dog remained in Zone 1 for 30 s without completing a nose-touch, including those blocks not eligible for reinforcement, the trainer tossed a treat into Zone 2 to encourage it to leave Zone 1 and then re-enter it for another trial. Training ended if it (a) did not enter Zone 1 within 2 min of its previous nose-touch; (b) completed the shaping phase and all 11 testing levels in less than 30 min; or (c) once 30 min had elapsed—whichever occurred first.

## Results and discussion

Data from 30 dogs in each group were analyzed. The assumptions of normality and homogeneity of variances of residuals were verified with a Shapiro–Wilk test (*W*(90) = 0.98, *p* = 0.12), a Levene’s test (*F*(2, 87) = 0.09, *p* = 0.91), and we confirmed that no standard deviation of one group exceeded twice the standard deviation of any other group (SD_primary_ = 2.7; SD_verbal_ = 2.4; SD_clicker_ = 3.0). A MANCOVA was used, instead of an ANCOVA as in Experiment 1 and 2, to account for testing levels nearest each other likely correlating more highly than levels further apart. Missing data on the nose-touches-per-level variable were handled using maximum likelihood estimation, which achieved maximum power compared to alternatives such as listwise deletion (Schafer & Graham, 2002). Because two dependent variables were assessed using the same dogs, a Bonferroni adjustment was used to control the family-wise Type I error rate for the two sets of tests, comparing results to a nominal alpha = 0.025. Although effect sizes and confidence intervals for pairwise differences are reported for descriptive purposes only, reported CIs are 97.5%, to be consistent with the Bonferroni adjustment.

Due to violation of the sphericity assumption, a one-factor MANCOVA was performed in SPSS (Version 25) International Business Machines Corp. (2017) controlling for age, sex, and weight to compare the influence of reinforcement group on the levels achieved. Figure 3C provides the means and standard errors of the means for each group. The MANCOVA indicated that the difference in number of levels achieved between groups was not statistically significant, *F*(2, 86) = 1.25, *p* = 0.29. The covariates of sex (*p* = 0.04), age (*p* = 0.14), and weight (*p* = 0.06) were also not statistically significant. Effect sizes for the three pairwise differences in the number of levels achieved were *d* = 0.35 (97.5% CI [−0.24 to 0.95]), *d* = 0.01 (97.5% CI [−0.57 to 0.59]), and *d* = 0.33 (97.5% CI [−0.25 to 0.91]), for comparing verbal secondary to primary alone, clicker secondary to primary alone, and clicker secondary to verbal secondary, respectively.

To test whether dogs in the clicker secondary group attained a different response rate at each level than those in the primary alone or verbal secondary groups, we performed a one-factor MANCOVA in SAS (Version 9.4; Cary, NC, USA) controlling for age, sex, and weight comparing the influence of group on the average number of nose-touches made per testing level. PROC MIXED, specifying a random intercept, was used to employ maximum likelihood estimation for missing data. Blind coders recorded the number of nose-touches made by all dogs from their video recordings. The video of one dog in the verbal secondary reinforcement group was lost and unable to be coded. Mean count-per-interval inter-observer agreement (IOA) was tested on 8 out of 30 videos in the primary alone and clicker secondary groups, and on 8 out of 29 videos in the verbal secondary group. IOA was above 90%. The MANCOVA indicated no statistically significant difference between groups in the average number of nose-touches made per level, *F*(2, 55.4) = 2.21, *p* = 0.12, and that the level-by-group interaction was also not significant, *F*(20, 37.8) = 0.59, *p* = 0.89, with no covariate reaching statistical significance, *p* = 0.80 for age, *p* = 0.34 for sex, and *p* = 0.20 for weight. There was a linear trend to the data when testing the relationship between number of nose-touches and testing level, *F*(1, 26.1) = 39.64, *p* < 0.01, indicating that dogs responded more frequently as testing level increased, controlling for age, sex, and weight. Effect sizes for the three pairwise differences in the average number of nose-touches made per level were *d* = 0.11 (97.5% CI [−0.48 to 0.69]), *d* = 0.31(97.5% CI [−0.28 to 0.89]), and *d* = 0.39 (97.5% CI [−0.20 to 0.97]), for comparing verbal secondary to primary alone, clicker secondary to primary alone, and clicker secondary to verbal secondary, respectively.

To investigate the possibility of differences in delay to reinforcement in our groups, we randomly selected 12 dogs from each group and blind coders analyzed the first, middle, and last 2-min segments of their videos and recorded the duration of their delays to primary reinforcement using BORIS (Version 5.1.0; Friard & Gamba, 2016). We found that the average delays to reinforcement per condition for the primary alone, verbal secondary, and clicker secondary groups were 1.2 s (SD = 0.6), 1.5 s (SD = 0.5), and 1.3 s (SD = 0.4) respectively. As group performance was ranked by the delay to reinforcement, it is possible that the delay to reinforcement affected the testing level achieved in each of the groups.

## Discussion

Across three experiments, we found no evidence to support the claim that using a clicker as a secondary reinforcer for training dogs results in acquisition of a novel behavior more rapidly or to a higher level compared to primary reinforcement alone or a verbal secondary reinforcer. The only significant difference between reinforcement conditions in our experiments was between the primary alone and verbal secondary groups in Experiment 1, where dogs in the primary alone group attained a significantly longer mean duration of a sit than verbally-reinforced dogs. These results have some points of consistency with both Thorn et al. (2006) and Dorey, Blandina & Udell (2020) in that neither study found clicker-trained dogs learned a sit behavior significantly faster than those trained with primary reinforcement alone or with a verbal secondary reinforcer. The absence of any significant differences between the primary reinforcement and either of the secondary reinforcement groups in both our second and third experiments is consistent with findings by Smith & Davis (2008), Chiandetti et al. (2016), Dorey, Blandina & Udell (2020), and Williams et al. (2004). Smith & Davis (2008) and Williams et al. (2004) found no difference between primary reinforcement alone and a clicker group when teaching Basenjis and horses respectively a targeting behavior, both using procedures similar to that reported in our second experiment. When teaching behaviors of greater specificity akin to that of our third experiment, Chiandetti et al. (2016) observed no benefit of either form of secondary reinforcement when training dogs to open a bread box, and neither did Dorey, Blandina & Udell (2020) when training dogs to perform a “wave” behavior.

To date, one study has suggested any benefit to a clicker secondary reinforcer. Paredes-Ramos et al. (2020) reported that clicker-trained piglets acquired a novel behavior in significantly fewer trials than did a verbally-reinforced group. A single trainer used a 10-step shaping protocol to teach ten piglets in each group to fetch a novel object for 30 trials a day until all piglets acquired the behavior. A piece of a packaged cookie was the primary reinforcer, which was placed on the ground 1 s after piglets heard their secondary reinforcer. Each day, a different object had to be fetched, and the criterion for the ten shaping levels alternated between five and three consecutive repetitions at every other shaping level before advancing to the next one. Piglets have received little if any prior attention from scientists studying the acquisition of novel behavior, so the particular combination of conditions that led to this result and its broader implications remain unclear.

In the following sections, we address possible explanations for the lack of support for greater efficacy of a clicker over other available reinforcement methods.

### Age of the dogs tested

It might be argued that the dogs in our studies were too young to attain the behaviors we attempted to train, however the success of a majority of dogs in each experiment contradicts this contention. We also included age as a covariate in all our analyses and did not find any significant impact on the dogs’ performance. At least one prior study found that dogs as young as 1.5 months can be successfully trained in operant tasks. Lozovskaia (1985) reported that 1.5-month-old pups learned an escape task more rapidly than 7-month-olds.

### Statistical power

Another possible explanation for our failure to find a positive impact of using a secondary reinforcer is that our studies did not have sufficient power to detect an effect. Our experiments only had enough power to detect medium and large effects, and thus a small effect of the clicker could have gone undetected. On the other hand, the lack of a clear clicker effect found in our (and others) studies, over several replications, provides evidence that if there is any effect, it may not be large enough to have practical impact under testing conditions explored to date.

### Number of primary reinforcement pairings

To test the effect of the clicker on the acquisition of a novel behavior, it must be established as a secondary reinforcer by pairing it sufficiently with the primary prior to testing. In our experiments, the clicker was paired with the primary reinforcer 20 times in order to establish the conditional relationship, which was consistent with both the Smith & Davis (2008) and Dorey, Blandina & Udell (2020) procedures in dogs and Williams et al. (2004) with horses. Under laboratory conditions, Skinner (1938) recommended between 30 (1951) and 60 (1938) pairings of the CS and US for dogs and rats, respectively, while Kelleher & Gollub (1962) indicated that pairings beyond 100 trials no longer strengthened the conditioned reinforcement effect for rats. On the other hand, Pryor (2006) has suggested that only two or three pairings are needed to establish the clicker as a conditioned reinforcer. Similarly, Chiandetti et al. (2016) claimed that two or three pairings were sufficient, and neither Thorn et al. (2006) nor Paredes-Ramos et al. (2020) paired the auditory stimulus with the primary reinforcer at all before initiating testing. While additional pairings would be expected to more strongly establish the stimulus as a secondary reinforcer (Wike, 1966), our procedure was consistent with or more substantial than others in the literature, and we were constrained by the possibility of satiation in our young subjects. It is unlikely that dogs tested in our experiments became satiated prior to the start of testing given the size of each primary reinforcer (approximately 0.63 cm^3^), and 83% of dogs tested across all experiments continued participating after the pairing protocol, but to provide more than 20 pieces of primary reinforcement before testing began could have affected the motivation of our subjects.

### Function of the clicker

Despite the widespread belief that clicker training facilitates faster acquisition of a novel behavior in dogs (Feng, Howell & Bennett, 2018), the peer-reviewed scientific literature has consistently shown that this is not the case. As Feng, Howell & Bennett (2016) and Dorey & Cox (2018) have noted, ambiguity exists in the definitions that scientists and practitioners use when referring to “clicker training,” which is concerning given the apparent disconnect in the two communities’ beliefs in the efficacy of clicker training. Martin & Friedman (2011) speculated that in clicker training, the “click” sound is a secondary reinforcer, a bridging stimulus, and an event marker. A marking stimulus is distinguished from a secondary reinforcer in that it does not provide information about a future opportunity to obtain primary reinforcement. Rather, it is simply a novel and unexpected auditory or visual cue that distinguishes the targeted response from the other behaviors the animal was emitting at that time (Lieberman, McIntosh & Thomas, 1979). If the clicker were a bridging stimulus, it would temporally connect the desired response with the delayed food reinforcement through stimulus-stimulus relationships as opposed to response-stimulus relationships (Dorey & Cox, 2018; Williams, 1994). As utilized in the testing phases of our and others’ experiments, the clicker was not a novel stimulus; however it did provide information about a future opportunity to obtain primary reinforcement, and it connected response-stimulus relationships. Although no study has directly tested if the clicker functions as a marking stimulus, bridging stimulus, or secondary reinforcer, by definition alone it does not seem as though the clicker could have been functioning as either a marking or bridging stimulus; instead, the clicker appears to function most similar to a secondary reinforcer because it is deployed immediately following the completion of the desired response and is paired to reliably predict the arrival of the primary reinforcer. Dorey & Cox (2018) suggested that the effectiveness of the clicker as a secondary reinforcer could be tested by presenting the clicker contingent upon the occurrence of a new response, or by comparing resistance to extinction for individuals in which a clicker was used in training and those for whom it was not. While the latter recommendation has been shown to be unreliable in measuring the strength of the secondary reinforcer in laboratory experiments with rats (Kelleher & Gollub, 1962; Williams, 1994), implementing a final testing phase in which the clicker is used to train a novel behavior in an applied setting could be used to detect evidence of the clicker’s effect as a secondary reinforcer (Wike, 1966), as was done in Smith & Davis (2008).

If the clicker was functioning as a secondary reinforcer, we would expect to see any additional reinforcement value of the clicker reflected in an increased rate of performing the desired behavior compared to a control group that only receives primary reinforcement (Williams, 1991). Although we did not find this effect here, standard learning theory states that in a situation in which the primary reinforcer is always promptly available, the influence of a secondary reinforcer on the rate of acquisition is weak compared to that of the primary reinforcer alone (Rescorla & Wagner, 1972).

Laboratory studies dating back to Pavlov (1928) have shown that that more rapid reinforcement leads to better acquisition, thus emphasizing the importance of measuring delays to reinforcement in studies of the efficacy of reinforcement methods. The three experiments reported here show somewhat contradictory findings on this issue, as the rank order of delay to reinforcement did not match performance in the first two experiments, but it did for the third experiment. No prior study comparing clicker training to other positive reinforcement methods has reported the delay to reinforcement for any condition, so the impact of adding a secondary reinforcer on behavioral acquisition in other scenarios is unknown. Clickers or other secondary reinforcers could improve acquisition of novel behavior by reducing the delay to reinforcement in situations where it is not possible to provide immediate primary reinforcement, but they might also have a negative impact on behavioral acquisition in situations where the provision of a secondary reinforcer increases the delay to primary reinforcement, such as when the trainer is already standing next to their dog. Future research should investigate this issue directly by experimentally manipulating the delays to secondary and primary reinforcement.

### Applied environment

In applied settings such as where dogs are typically trained, control of environmental stimuli is much less precise and variability in testing conditions is inevitably introduced by the presence of a human in the environment, which leads to uncontrolled factors involved in the relationship of the animal with the human as well as errors in the timing and delivery of stimuli. Our studies and those of Thorn et al. (2006), Smith & Davis (2008), Chiandetti et al. (2016), and Dorey, Blandina & Udell (2020) attempted to maintain controlled experimental environments similar to a laboratory setting, but the environments of these studies are closer in actuality to the real world than a typical animal laboratory. It is quite possible that clicker training functions differently in applied settings than in laboratories, and interactions between dogs and their trainers must play a role in learning (Feng, Howell & Bennett, 2018; Pryor, 1999).

Feng et al. (2018) attempted to evaluate the effectiveness of clickers in the home setting by asking owners to teach their dogs new behaviors using either a clicker followed by food or delivering primary reinforcement only. After training, owners were asked to rate their dogs’ and their own perceived difficulty and enjoyment of the task. Feng et al. (2018) found that owners in the clicker group reported a less challenging training experience when teaching a nose-targeting behavior than was reported by owners in the food-only group but observed no differences between training groups for the other five behaviors. No measures were taken of the dogs’ success in learning the different tasks. It is possible that more robust inferences about the training methods could have been made had owners trained their dogs with both methods and then rated the perceived difficulty and enjoyment of each method, or if the dogs’ performances when learning these new behaviors had been objectively measured.

Real-world environments also involve a great diversity of individuals attempting to train their dogs, and future studies should also investigate the impact of variability among trainers on behavioral outcomes. The current study, in line with Thorn et al. (2006), Smith & Davis (2008), Chiandetti et al. (2016), and Dorey, Blandina & Udell (2020) only utilized one trainer throughout testing. This is an advantage for assessment of the impact of a training method but should be broadened in future studies. China, Mills & Cooper (2020), in a study comparing positive reinforcement to positive punishment in the training of dogs, found significant differences in behavioral acquisition between professional trainers utilizing the same testing protocol.

## Conclusions

Of the three experiments reported here investigating the circumstances under which a clicker may result in acquisition of a novel behavior more rapidly or to a higher level of difficulty or specificity compared to other readily available reinforcement methods, only one difference between groups was detected. In Experiment 1, dogs trained to sit for increasing periods of time with primary reinforcement alone reached a significantly higher level of performance than those trained with verbal secondary reinforcement. Clearly, in the present experiments, we have only explored a small subset of the ways in which clickers could be used in dog training. Clickers can certainly be used to train far more complex behaviors than what was studied here—the SPCA Auckland used clicker training to teach dogs how to drive a car (Wynne, 2012)—but thus far there is no evidence that such behaviors could not be as efficiently trained using any other reinforcement method. Future investigations should explore how clicker-trained dogs perform on different tasks, with special focus on the role of delay to secondary and primary reinforcement, and how clicker training functions in an applied setting, bearing in mind the need for objective outcome measures which are independent of owners’ and trainers’ preconceptions.

## Supplemental Information

10.7717/peerj.10881/supp-1 Supplemental Information 1 Complete dataset for Experiments 1, 2, and 3 detailing trial-by-trial performance for all dogs that completed at least one trial. Each A# represents an individual dog, with their performance on each trial reflected in the column below their identifying number. Dogs in Experiments 1 and 2 could complete no more than 50 trials, while dogs in Experiment 3 could complete no more than 49 trials.

10.7717/peerj.10881/supp-2 Supplemental Information 2 Dogs that participated in Experiment 1. “Longest sit” is the duration in seconds of the longest sit achieved in Experiment 1, except for dogs noted as “sat on command,” or “never sat.” Seven dogs were excluded for sitting on first instruction; 13 for not sitting within 25 minutes of beginning training; and one due to identification of illness. Sex is male (m) or female (f). Age, sex, and weight were determined at the date the dogs were tested. IDs are those noted in shelter records. A dog for which the identification number was not recorded at the time of testing is missing its weight measurement and as such is marked “unknown.”

10.7717/peerj.10881/supp-3 Supplemental Information 3 Dogs that participated in Experiment 2. “Highest Step” is the number of the highest completed training step achieved during the shaping and distance components of Experiment 2, except for dogs noted as “never touched.” Sex is male (m) or female (f). Age, sex, and weight were determined on the date the dogs were tested. IDs are those noted in shelter records.

10.7717/peerj.10881/supp-4 Supplemental Information 4 Dogs that participated in Experiment 3. “Highest Level” is the number of the highest level of training the dog completed during testing in Experiment 3. Sex is male (m) or female (f). Age, sex, and weight were determined on the date dogs were tested. IDs are those noted in shelter records.

## Additional Information and Declarations

### Data Availability

The following information was supplied regarding data availability: Raw data is available in the Supplemental Files.
