# Supplementary material for: The click is not the trick: the efficacy of clickers and other reinforcement methods in training naïve dogs to perform new tasks
Source: PeerJ. 2021 Feb 22;9:e10881. doi: 10.7717/peerj.10881 (PMC7906040; doi:10.7717/peerj.10881)
Supplement: Supplemental Information 2 — “Longest sit” is the duration in seconds of the longest sit achieved in Experiment 1, except for dogs noted as “sat on command,” or “never sat.” Seven dogs were excluded for sitting on first instruction; 13 for not sitting within 25 minutes of beginning training; and one due to identification of illness. Sex is male (m) or female (f). Age, sex, and weight were determined at the date the dogs were tested. IDs are those noted in shelter records. A dog for which the identification number was not recorded at the time of testing is missing its weight measurement and as such is marked “unknown.” [file peerj-09-10881-s002.docx]

| Dog ID | Age (weeks) | Sex | Weight (kgs) | Condition | Longest Sit (s) |
| --- | --- | --- | --- | --- | --- |
| A#512945 | 10 | f | 2.04 | Primary alone | 16 |
| A#517844 | 9 | f | 3.76 | Primary alone | 34 |
| A#518272 | 13 | f | 1.59 | Primary alone | 7 |
| A#515400 | 11 | m | 9.07 | Primary alone | 7 |
| A#518461 | 17 | m | 3.76 | Primary alone | 28 |
| A#518833 | 12 | f | 5.31 | Primary alone | 31 |
| A#518830 | 12 | m | 4.72 | Primary alone | 13 |
| A#519080 | 21 | m | 13.29 | Primary alone | 37 |
| A#517692 | 23 | f | 3.08 | Primary alone | 7 |
| A#522622 | 17 | f | 4.26 | Primary alone | 10 |
| A#522562 | 11 | f | 4.44 | Primary alone | 25 |
| A#523272 | 11 | f | 4.54 | Primary alone | 40 |
| A#523471 | 9 | f | 3.31 | Primary alone | 1 |
| A#518704 | 11 | m | 2.00 | Primary alone | 22 |
| A#518705 | 11 | m | 1.81 | Primary alone | 13 |
| A#523473 | 9 | f | 3.22 | Primary alone | 28 |
| A#518703 | 11 | m | 2.27 | Primary alone | 25 |
| A#523855 | 21 | f | 5.26 | Primary alone | 25 |
| A#523657 | 8 | f | 1.81 | Primary alone | 25 |
| A#523854 | 21 | f | 5.26 | Primary alone | 16 |
| A#525318 | 20 | m | 9.98 | Primary alone | 25 |
| A#525436 | 11 | f | 2.04 | Primary alone | 1 |
| A#524641 | 11 | m | 2.18 | Primary alone | 1 |
| A#526451 | 21 | m | 5.62 | Primary alone | 16 |
| A#526850 | 14 | m | 18.42 | Primary alone | 19 |
| A#527128 | 22 | m | 11.25 | Primary alone | 1 |
| A#527246 | 9 | f | 2.81 | Primary alone | 13 |
| A#525159 | 8 | f | 3.67 | Primary alone | 4 |
| A#527361 | 10 | m | 0.95 | Primary alone | 1 |
| A#518213 | 11 | m | 3.18 | Primary alone | never sat |
| A#unknown | 12 | f | Unknown | Primary alone | sat on command |
| A#518895 | 11 | f | 1.95 | Primary alone | never sat |
| A#524100 | 18 | m | 13.65 | Primary alone | ill during testing |
| A#517731 | 11 | m | 3.63 | Verbal | never sat |
| A#517791 | 11 | f | 1.90 | Verbal | 13 |
| A#512943 | 11 | m | 1.95 | Verbal | 13 |
| A#517032 | 11 | f | 6.58 | Verbal | sat on command |
| A#518949 | 10 | f | 2.09 | Verbal | 13 |
| A#519408 | 22 | m | 19.05 | Verbal | 16 |
| A#517323 | 10 | f | 3.90 | Verbal | 10 |
| A#521748 | 10 | m | 1.77 | Verbal | 10 |
| A#516153 | 9 | m | 6.71 | Verbal | 1 |
| A#518960 | 20 | m | 30.50 | Verbal | 1 |
| A#521492 | 11 | f | 2.90 | Verbal | 1 |
| A#516533 | 10 | m | 3.22 | Verbal | 1 |
| A#517732 | 10 | m | 3.67 | Verbal | never sat |
| A#519369 | 8 | m | 1.54 | Verbal | 1 |
| A#517324 | 10 | m | 4.17 | Verbal | 1 |
| A#521997 | 13 | m | 3.81 | Verbal | 7 |
| A#519029 | 22 | m | 15.65 | Verbal | 7 |
| A#519719 | 9 | f | 1.22 | Verbal | 7 |
| A#518225 | 17 | m | 12.02 | Verbal | 10 |
| A#519756 | 9 | m | 2.27 | Verbal | 4 |
| A#521621 | 11 | f | 9.03 | Verbal | 16 |
| A#519733 | 20 | m | 7.70 | Verbal | 4 |
| A#523585 | 16 | m | 4.90 | Verbal | 37 |
| A#524035 | 13 | m | 3.63 | Verbal | 16 |
| A#525907 | 11 | m | 9.43 | Verbal | 7 |
| A#525320 | 20 | m | 9.52 | Verbal | 31 |
| A#525249 | 10 | m | 4.35 | Verbal | 19 |
| A#526852 | 9 | m | 3.67 | Verbal | 1 |
| A#527133 | 13 | f | 4.04 | Verbal | 4 |
| A#527247 | 9 | f | 2.81 | Verbal | 1 |
| A#524619 | 12 | f | 1.86 | Verbal | 1 |
| A#525199 | 11 | f | 5.26 | Verbal | 7 |
| A#526907 | 8 | m | 2.09 | Verbal | 7 |
| A#525158 | 11 | f | 4.13 | Verbal | never sat |
| A#527363 | 10 | f | 1.09 | Verbal | never sat |
| A#518831 | 12 | m | 5.40 | Verbal | never sat |
| A#523932 | 18 | m | 2.81 | Verbal | never sat |
| A#518035 | 22 | f | 3.13 | Verbal | never sat |
| A#522165 | 11 | f | 6.12 | Verbal | sat on command |
| A#518981 | 12 | f | 6.80 | Verbal | sat on command |
| A#518059 | 9 | f | 3.58 | Clicker | 7 |
| A#518463 | 22 | m | 3.54 | Clicker | 31 |
| A#518834 | 12 | m | 7.85 | Clicker | 31 |
| A#518950 | 10 | f | 2.54 | Clicker | 13 |
| A#519720 | 9 | m | 1.32 | Clicker | 19 |
| A#521027 | 11 | m | 6.94 | Clicker | 28 |
| A#522013 | 16 | f | 5.12 | Clicker | 37 |
| A#521749 | 10 | m | 1.45 | Clicker | 13 |
| A#521689 | 9 | m | 1.86 | Clicker | 1 |
| A#519188 | 18 | m | 7.89 | Clicker | 1 |
| A#517834 | 13 | m | 4.94 | Clicker | 1 |
| A#519368 | 8 | f | 1.50 | Clicker | 4 |
| A#518273 | 9 | f | 3.26 | Clicker | 1 |
| A#521751 | 9 | m | 1.41 | Clicker | 1 |
| A#521492 | 10 | f | 2.90 | Clicker | 10 |
| A#519718 | 9 | f | 1.59 | Clicker | 13 |
| A#518212 | 12 | f | 16.01 | Clicker | 31 |
| A#523415 | 13 | m | 2.81 | Clicker | 7 |
| A#523853 | 21 | f | 5.99 | Clicker | 10 |
| A#524101 | 22 | f | 11.34 | Clicker | 1 |
| A#524036 | 13 | m | 4.40 | Clicker | 13 |
| A#525794 | 13 | m | 10.43 | Clicker | 1 |
| A#525434 | 11 | m | 3.04 | Clicker | 13 |
| A#526779 | 12 | f | 3.45 | Clicker | 4 |
| A#526775 | 12 | f | 2.00 | Clicker | never sat |
| A#526851 | 9 | f | 2.72 | Clicker | 19 |
| A#527132 | 13 | m | 3.00 | Clicker | 28 |
| A#527248 | 9 | m | 3.22 | Clicker | 16 |
| A#527428 | 11 | f | 4.22 | Clicker | 13 |
| A#525201 | 11 | f | 4.99 | Clicker | never sat |
| A#525197 | 11 | f | 4.35 | Clicker | never sat |
| A#526906 | 8 | m | 2.18 | Clicker | 1 |
| A#528039 | 9 | m | 3.90 | Clicker | 22 |
| A#521750 | 9 | m | 1.27 | Clicker | never sat |
| A#521244 | 10 | m | 8.07 | Clicker | sat on command |
| A#520198 | 16 | m | 6.44 | Clicker | sat on command |
| A#518621 | 22 | f | 6.89 | Clicker | sat on command |
