# Supplementary material for: The click is not the trick: the efficacy of clickers and other reinforcement methods in training naïve dogs to perform new tasks
Source: PeerJ. 2021 Feb 22;9:e10881. doi: 10.7717/peerj.10881 (PMC7906040; doi:10.7717/peerj.10881)
Supplement: Supplemental Information 3 — “Highest Step” is the number of the highest completed training step achieved during the shaping and distance components of Experiment 2, except for dogs noted as “never touched.” Sex is male (m) or female (f). Age, sex, and weight were determined on the date the dogs were tested. IDs are those noted in shelter records. [file peerj-09-10881-s003.docx]

| Dog ID | Age (weeks) | Sex | Weight (kg) | Condition | Highest Step |
| --- | --- | --- | --- | --- | --- |
| A#537279 | 10 | m | 1.5 | primary alone | 8 |
| A#537649 | 15 | f | 10.9 | primary alone | 8 |
| A#537712 | 17 | m | 10.9 | primary alone | 16 |
| A#537189 | 9 | m | 2.7 | primary alone | 7 |
| A#538221 | 18 | m | 2.6 | primary alone | 11 |
| A#538598 | 11 | m | 3.3 | primary alone | 5 |
| A#536320 | 11 | m | 5.3 | primary alone | 17 |
| A#535787 | 9 | m | 5.5 | primary alone | 12 |
| A#535759 | 10 | f | 5.6 | primary alone | 8 |
| A#539316 | 13 | m | 10.4 | primary alone | 7 |
| A#539885 | 10 | m | 4.5 | primary alone | 2 |
| A#538378 | 10 | f | 4.4 | primary alone | 11 |
| A#537506 | 10 | m | 4.6 | primary alone | 8 |
| A#539676 | 16 | m | 4.0 | primary alone | never touched |
| A#539190 | 11 | f | 1.6 | primary alone | 5 |
| A#540279 | 11 | m | 3.2 | primary alone | 5 |
| A#539987 | 11 | f | 2.2 | primary alone | 5 |
| A#541156 | 22 | f | 10.7 | primary alone | 7 |
| A#539182 | 8 | m | 3.4 | primary alone | 9 |
| A#540922 | 18 | f | 7.1 | primary alone | 7 |
| A#541049 | 9 | m | 2.0 | primary alone | 4 |
| A#540307 | 13 | f | 7.3 | primary alone | 8 |
| A#542066 | 9 | f | 2.0 | primary alone | 6 |
| A#542031 | 8 | f | 2.3 | primary alone | 7 |
| A#539026 | 9 | m | 2.3 | primary alone | 9 |
| A#541381 | 9 | m | 2.5 | primary alone | 9 |
| A#537673 | 9 | f | 2.6 | primary alone | never touched |
| A#538443 | 12 | f | 3.5 | primary alone | never touched |
| A#537412 | 10 | m | 1.7 | verbal | 8 |
| A#537621 | 8 | f | 2.3 | verbal | 8 |
| A#535399 | 9 | m | 6.2 | verbal | 6 |
| A#535400 | 9 | m | 5.8 | verbal | never touched |
| A#537405 | 22 | f | 1.9 | verbal | 5 |
| A#535794 | 13 | f | 3.1 | verbal | 5 |
| A#539172 | 14 | m | 8.9 | verbal | 7 |
| A#535754 | 9 | m | 6.1 | verbal | 13 |
| A#534018 | 22 | f | 6.1 | verbal | 13 |
| A#539391 | 20 | m | 9.2 | verbal | 5 |
| A#538383 | 13 | m | 4.5 | verbal | 8 |
| A#538414 | 15 | f | 13.2 | verbal | 8 |
| A#538382 | 14 | m | 5.4 | verbal | 8 |
| A#537503 | 10 | m | 4.2 | verbal | 3 |
| A#540550 | 8 | f | 5.2 | verbal | 8 |
| A#539986 | 11 | f | 2.3 | verbal | 7 |
| A#541157 | 22 | f | 10.2 | verbal | 7 |
| A#539183 | 9 | m | 4.5 | verbal | 8 |
| A#540923 | 18 | m | 6.9 | verbal | 15 |
| A#541034 | 22 | f | 3.4 | verbal | 6 |
| A#541047 | 9 | f | 1.6 | verbal | 7 |
| A#541048 | 9 | m | 2.1 | verbal | 3 |
| A#541733 | 18 | f | 1.5 | verbal | 1 |
| A#542030 | 8 | f | 1.9 | verbal | 15 |
| A#541147 | 22 | f | 7.5 | verbal | 6 |
| A#541380 | 9 | f | 2.5 | verbal | 5 |
| A#530751 | 19 | m | 2.8 | verbal | never touched |
| A#539194 | 9 | m | 2.0 | verbal | never touched |
| A#539886 | 11 | f | 5.2 | verbal | never touched |
| A#531904 | 11 | f | 6.9 | clicker | 13 |
| A#537963 | 10 | m | 6.8 | clicker | 9 |
| A#536715 | 11 | m | 4.6 | clicker | 1 |
| A#538483 | 11 | f | 1.5 | clicker | 7 |
| A#538219 | 9 | m | 2.0 | clicker | 10 |
| A#538597 | 10 | m | 4.4 | clicker | 8 |
| A#535790 | 9 | m | 5.4 | clicker | 16 |
| A#535753 | 9 | m | 6.8 | clicker | 1 |
| A#535793 | 10 | f | 5.2 | clicker | 6 |
| A#539545 | 9 | m | 5.7 | clicker | 14 |
| A#539886 | 9 | f | 5.2 | clicker | 1 |
| A#540061 | 16 | f | 9.9 | clicker | 14 |
| A#539762 | 11 | f | 2.6 | clicker | 10 |
| A#537502 | 10 | m | 4.6 | clicker | 4 |
| A#540284 | 11 | f | 3.0 | clicker | 11 |
| A#541070 | 22 | m | 16.4 | clicker | 8 |
| A#539184 | 9 | f | 3.7 | clicker | 9 |
| A#540921 | 18 | m | 7.3 | clicker | 8 |
| A#537179 | 11 | m | 5.9 | clicker | 17 |
| A#541046 | 9 | f | 1.8 | clicker | 4 |
| A#541715 | 10 | m | 1.9 | clicker | 7 |
| A#542029 | 8 | f | 1.9 | clicker | 8 |
| A#541146 | 22 | f | 9.8 | clicker | 12 |
| A#540981 | 11 | f | 2.6 | clicker | 7 |
| A#540982 | 11 | m | 2.5 | clicker | 7 |
| A#537857 | 10 | m | 1.8 | clicker | never touched |
