# Supplementary material for: The click is not the trick: the efficacy of clickers and other reinforcement methods in training naïve dogs to perform new tasks
Source: PeerJ. 2021 Feb 22;9:e10881. doi: 10.7717/peerj.10881 (PMC7906040; doi:10.7717/peerj.10881)
Supplement: Supplemental Information 4 — “Highest Level” is the number of the highest level of training the dog completed during testing in Experiment 3. Sex is male (m) or female (f). Age, sex, and weight were determined on the date dogs were tested. IDs are those noted in shelter records. [file peerj-09-10881-s004.docx]

| Dog ID | Age (weeks) | Sex | Weight (kgs) | Condition | Highest Level |
| --- | --- | --- | --- | --- | --- |
| A#550782 | 13 | m | 2.95 | primary alone | 9 |
| A#552544 | 9 | f | 1.63 | primary alone | 4 |
| A#552246 | 8 | f | 3.81 | primary alone | 2 |
| A#551316 | 11 | f | 2.68 | primary alone | 10 |
| A#548848 | 11 | f | 1.95 | primary alone | 10 |
| A#552967 | 10 | f | 4.58 | primary alone | 11 |
| A#553752 | 14 | m | 3.67 | primary alone | 7 |
| A#551396 | 11 | m | 1.95 | primary alone | 11 |
| A#554444 | 10 | f | 2.77 | primary alone | 11 |
| A#554855 | 10 | m | 3.67 | primary alone | 3 |
| A#554843 | 14 | f | 5.76 | primary alone | 11 |
| A#553355 | 19 | f | 8.84 | primary alone | 9 |
| A#551789 | 11 | f | 2.00 | primary alone | 6 |
| A#555534 | 9 | m | 1.91 | primary alone | 7 |
| A#552842 | 8 | f | 2.27 | primary alone | 7 |
| A#555477 | 9 | f | 2.99 | primary alone | 8 |
| A#555473 | 9 | m | 2.49 | primary alone | 9 |
| A#553347 | 11 | m | 5.22 | primary alone | 8 |
| A#555389 | 22 | f | 3.40 | primary alone | 8 |
| A#555978 | 9 | m | 3.58 | primary alone | 6 |
| A#552657 | 9 | m | 6.80 | primary alone | 11 |
| A#552659 | 9 | m | 7.26 | primary alone | 9 |
| A#566337 | 9 | f | 4.13 | primary alone | 7 |
| A#569018 | 9 | m | 1.32 | primary alone | 7 |
| A#564175 | 12 | m | 0.91 | primary alone | 3 |
| A#566396 | 10 | f | 1.32 | primary alone | 5 |
| A#569256 | 8 | f | 1.09 | primary alone | 3 |
| A#569567 | 22 | m | 6.98 | primary alone | 11 |
| A#568235 | 8 | m | 1.63 | primary alone | 9 |
| A#569189 | 10 | f | 4.81 | primary alone | 7 |
| A#554800 | 9 | m | 2.45 | primary alone | did not eat |
| A#553304 | 8 | m | 5.26 | primary alone | never touched |
| A#552239 | 10 | f | 5.44 | primary alone | no shaping |
| A#552959 | 9 | m | 4.81 | primary alone | no shaping |
| A#553627 | 11 | f | 3.99 | primary alone | no shaping |
| A#549469 | 12 | f | 1.90 | primary alone | no shaping |
| A#551780 | 10 | m | 2.09 | verbal | 6 |
| A#552696 | 11 | f | 1.59 | verbal | 7 |
| A#552244 | 8 | f | 4.31 | verbal | 2 |
| A#549468 | 11 | f | 2.00 | verbal | 6 |
| A#553167 | 12 | m | 1.77 | verbal | 9 |
| A#552237 | 10 | f | 4.99 | verbal | 7 |
| A#553753 | 8 | f | 4.67 | verbal | 5 |
| A#554106 | 13 | f | 6.80 | verbal | 7 |
| A#554856 | 10 | f | 2.86 | verbal | 4 |
| A#554517 | 12 | m | 4.44 | verbal | 9 |
| A#555011 | 22 | f | 13.42 | verbal | 7 |
| A#553333 | 8 | f | 1.81 | verbal | 3 |
| A#555698 | 10 | f | 5.35 | verbal | 8 |
| A#554123 | 12 | m | 2.31 | verbal | 10 |
| A#552840 | 8 | m | 2.81 | verbal | 11 |
| A#555475 | 9 | f | 2.77 | verbal | 5 |
| A#555472 | 10 | m | 2.99 | verbal | 2 |
| A#550992 | 13 | m | 2.77 | verbal | 7 |
| A#553332 | 11 | m | 1.68 | verbal | 4 |
| A#552658 | 9 | m | 6.58 | verbal | 7 |
| A#566342 | 9 | m | 3.63 | verbal | 6 |
| A#566335 | 9 | f | 4.44 | verbal | 7 |
| A#568217 | 8 | m | 1.68 | verbal | 10 |
| A#564176 | 12 | f | 1.04 | verbal | 6 |
| A#568639 | 22 | m | 2.77 | verbal | 3 |
| A#568596 | 10 | f | 1.63 | verbal | 7 |
| A#568957 | 10 | m | 2.63 | verbal | 8 |
| A#568958 | 10 | m | 2.63 | verbal | 10 |
| A#569979 | 18 | m | 2.40 | verbal | 9 |
| A#569187 | 10 | m | 5.49 | verbal | 8 |
| A#551478 | 13 | f | 3.36 | verbal | never touched |
| A#550990 | 11 | m | 2.27 | verbal | never touched |
| A#568832 | 14 | m | 10.43 | verbal | vomited |
| A#569051 | 11 | f | 3.22 | verbal | knew touch |
| A#552960 | 9 | f | 4.76 | verbal | no shaping |
| A#552970 | 10 | f | 4.13 | verbal | no shaping |
| A#552968 | 10 | f | 3.18 | verbal | no shaping |
| A#553630 | 11 | f | 4.08 | verbal | no shaping |
| A#555208 | 17 | m | 8.39 | verbal | no shaping |
| A#552656 | 9 | m | 6.80 | verbal | no shaping |
| A#565565 | 8 | f | 3.36 | verbal | never touched |
| A#550228 | 11 | m | 2.40 | clicker | 11 |
| A# 551477 | 13 | f | 3.76 | clicker | 5 |
| A#548691 | 12 | f | 3.31 | clicker | 7 |
| A#552242 | 8 | f | 4.54 | clicker | 8 |
| A#548849 | 13 | f | 1.72 | clicker | 4 |
| A#552958 | 9 | f | 3.08 | clicker | 7 |
| A#553956 | 14 | f | 2.86 | clicker | 11 |
| A#552966 | 10 | f | 3.04 | clicker | 3 |
| A#553955 | 14 | f | 3.31 | clicker | 11 |
| A#553741 | 10 | m | 9.25 | clicker | 11 |
| A#554858 | 10 | m | 3.13 | clicker | 4 |
| A#554857 | 10 | m | 3.13 | clicker | 10 |
| A#555036 | 21 | f | 10.16 | clicker | 10 |
| A#554668 | 18 | m | 13.24 | clicker | 9 |
| A#554903 | 18 | f | 7.35 | clicker | 11 |
| A#553328 | 8 | f | 2.22 | clicker | 2 |
| A#555381 | 15 | m | 6.62 | clicker | 3 |
| A#552841 | 8 | m | 1.81 | clicker | 10 |
| A#555476 | 8 | f | 2.77 | clicker | 3 |
| A#555474 | 10 | f | 2.99 | clicker | 7 |
| A#555390 | 22 | m | 5.17 | clicker | 11 |
| A#553329 | 11 | f | 2.68 | clicker | 5 |
| A#552655 | 9 | f | 5.67 | clicker | 6 |
| A#565562 | 8 | m | 3.45 | clicker | 7 |
| A#568048 | 22 | f | 9.16 | clicker | 9 |
| A#569010 | 18 | f | 16.33 | clicker | 11 |
| A#569403 | 22 | m | 12.70 | clicker | 11 |
| A#568239 | 8 | f | 1.54 | clicker | 6 |
| A#569180 | 10 | m | 6.26 | clicker | 7 |
| A#569195 | 10 | m | 6.35 | clicker | 7 |
| A#555702 | 17 | m | 12.61 | clicker | never touched |
| A#553751 | 8 | m | 4.08 | clicker | no shaping |
| A#555212 | 17 | f | 8.66 | clicker | no shaping |
| A#554762 | 11 | m | 4.76 | clicker | no shaping |
| A#565567 | 8 | f | 2.90 | clicker | no shaping |
